# Influences of Low Intensity on Diode Parameters of CdTe Solar Cells

**DOI:** 10.3390/ma13092194

**Published:** 2020-05-10

**Authors:** Xiaobo Xu, Wenping Gu, Xiaoyan Wang, Wei Zhu, Lin Zhang, Zan Zhang

**Affiliations:** School of Electronic and Control Engineering, Chang’an University, Xi’an 710064, China; wpgu@chd.edu.cn (W.G.); wxyswallow7907@163.com (X.W.); wzhu@chd.edu.cn (W.Z.); zhanglin_dk@chd.edu.cn (L.Z.); z.zhang@chd.edu.cn (Z.Z.)

**Keywords:** CdTe, solar cell, low intensity

## Abstract

This study deals with the CdS/CdTe solar cells under low illumination intensity, with cell #1 for the shunt resistance exceeding 100,000 Ω·cm^2^ and cell #2 for the shunt resistance above 1000 Ω·cm^2^. The diode parameter variations with the decline of the irradiance intensity are illustrated by dividing 0–100 mW/cm^−2^ into a number of small intensity ranges for J–V measurements and assuming the diode parameters to be constant within each range, the diode parameters of each range including the series resistance, the shunt resistance, the reverse saturation current density and the ideality factor are then extracted by employing an analytical approach. The mechanism of the cell performance deviations are also investigated by basic theories, reports and experiments. For cell #1 with higher *R_sh_* corresponding to less traps, *R_sh_* shows a upward tendency as the irradiance declines, *n* and *J*_0_ exhibit a rise with the irradiance and keep nearly unchanged at the low irradiance values mainly due to recombination and carrier contributions, *R_s_* shows a slight increase when the irradiance intensity goes down because of the resistance of CdTe absorption layer. For cell #2 with lower *R_sh_* corresponding to more traps, with the decrease of the illumination intensity, *R_sh_* increases sharply only for captured carrier reduction, *R_s_* goes steadily up similarly, *n* and *J*_0_ exhibit a decline with the irradiance due to recombination shift. It should be pointed out that *R_s_* varies much smoother than the traditional approximation of a reciprocal of differential at short circuit, and the distribution of *R_sh_* is diverse, and an average *R_sh_* of for each intensity range can reflect the variation trend.

## 1. Introduction

CdTe has established itself as the most promising material for thin film solar cell application on the market due to its direct bandgap with the value of about 1.45 eV close to the maximum of the solar spectrum the absorption coefficient of about 10^4^–10^5^ cm^−1^ in the visual part of the solar spectrum. Recent state-of-the-art CdTe solar cell has been fabricated in the lab with the efficiency surpassing that of polycrystalline Si at over 22% [1,2]. Most reports about CdTe solar cells concentrate on standard test condition (STC) with the illumination intensity of 100 mW/cm^2^ with a spectral distribution conforming to AM 1.5 spectrum and a cell temperature of 25 °C. However, these disagree with practical working conditions, by ignoring the case of rain, cloud, evening, morning, shade, etc., which deteriorate the irradiance intensity, so that the usefulness and applicability of the indoors’ characterization in STC of the solar cells are a controversial issue [3]. In fact, the intensity is normally lower than 100 mW/cm^2^, as confirmed by the data of the National renewable energy lab (NREL) [4] and the short circuit current density *J_sc_*, the open voltage *V_oc_*, the fill factor *FF*, and the conversion efficiency *η* depend on the irradiance, therefore it is essential to investigate the relationship between the diode parameters and the intensity. During previous reports about CdTe solar cells with low intensity, Randall et al. showed the comparisons of various solar cells [5,6], but the thorough analysis of the mechanism was missing, Shen et al. discussed the performance deviation with light irradiance in detail [7], and the ideality factor *n* and the reverse saturation current density *J*_0_ were assumed to be constant for simplicity, the series and shunt resistances were obtained by the reciprocal of differential at points of open and short circuits in the J–V curve, which are apparently not practical. Other researchers yield average values of *n*, *J*_0_, *R_s_* and *R_sh_* ignoring influences of the irradiance [8,9], except the work by D. L. Batzner et al. [10], which was mainly the comparison of different types of solar cells about key electrical feathers, although the irradiance dependence was plotted, the corresponding mechanism was not clearly demonstrated, furthermore, the extraction algorithm of *n*, *J*_0_, *R_s_* and *R_sh_* should be modified to improve the extraction accuracy. As *n* and *J*_0_ vary with the illumination intensity [11,12], the extraction methods of *R_s_* and *R_sh_* should also be more carefully built and selected. In this work, for CdTe solar cells, the parameter variation trends of *n*, *J*_0_, *R_s_* and *R_sh_* with illumination intensities are discussed, and the corresponding underlying mechanisms of carrier transportation and recombination are analyzed. Specifically, the cell parameters are determined from the combination of J–V curves at short circuit and open circuit within a small range of the intensities, in the way of linear fit and intersection with y axis. The data are from the CdTe module deployed on the Performance and Energy Ratings Testbed (PERT) system at the Outdoor Test Facility (OTF) [13], named cell #1, which is fabricated mainly by close-space sublimation deposition from a developmental deposition system [14]. The substrate is preheated to 400–600 °C, CdS is deposited with substrate temperature up to 450 °C, at a pressure of 1 Torr, and CdTe deposition follows the previous step with temperature up to 550 °C, the CdCl_2_ treatment is then applied. Cell #1 was normalized to characterize a cell in this study, in order to obtain meaningful ideality factor values between 1 and 2. Cell #2 is from the fabricated device in our lab with a similar fabrication process, with glass/SnO2/CdS/CdTe as the structure. The CdS layer was formed by chemical bath deposition (CBD) technique from cadmium acetate, ammonium acetate, thiourea and deionized water, and then heat treated in a container full of N_2_ with CdCl_2_ at the bottom. The polycrystalline CdTe absorber layer is prepared by close-spaced sublimation (CSS) with halogen lamp as the heating source, and graphite sheet as the substrate and then heat treated with a CdCl_2_ thin layer on the CdTe surface.

## 2. Model

The cell parameter estimation problem can be easily translated into an optimization problem, as the metaheuristic algorithms about soft computing could not guarantee consistency in computational and convergence speed, such as GA [15,16,17], PSO [18], etc., the analytical methods are thus employed because of its speed and simplicity. By omitting some minor factors, the nonlinear equation collapses to analytically solvable model, assessing the parameters directly. Among the analytical approaches, the algorithm based on two, three or more J–V curves under different levels of illumination shows good accuracy [8,19,20], which is employed by us. The detail is as follow.

The ideal single-diode J–V model of a solar cell can be represented as

(1) $$J=-J_{ph}+J_{0}\left(\exp\left(\frac{q(V-JR_{s})}{nkT}\right)-1\right)+\frac{V-JR_{s}}{R_{sh}}$$

where *J_ph_* and *J*_0_ are the photon and reverse current densities, respectively, *q* is elementary charge, *k* is Boltzmann constant, *T* is temperature in Kelvin, *n* is ideality factor, *R_s_* and *R_sh_* are series and shunt resistances, respectively. From the open (*J* = 0, *V* = *V_oc_*) and short (*V* = 0, *J* = −*J_SC_*) circuit conditions, the following models are derived based on the assumptions of *V_oc_* >> *J_sc_R_s_* and *R_sh_* >> *R_s_* [9].

(2) $$V_{oc}=\frac{nkT}{q}\ln\left(J_{sc}-\frac{V_{oc}}{m_{sc}^{-1}}\right)-\frac{nkT}{q}\ln\left(J_{0}\right)$$

(3) $$m_{oc}{}^{-1}=R_{s}+\frac{nkT}{qJ_{0}}\exp\left(\frac{-qV_{oc}}{nkT}\right)$$

where *m_sc_* and *m_oc_* are denoted as the differentiation of Equation (1) at short and open circuit. The lines of *V_oc_* versus ln(*J_sc_* − *V_oc_*/ *m_sc_*^−1^) and *m_oc_*^−1^ versus exp(−*qV_OC_*/(*nkT*)) can be fitted from the experimental values of *V_oc_*, *J_sc_* and *m_sc_*^−1^, demonstrated later. dJ/dV at short circuit condition approximately equals the reciprocal of *R_sh_*.

**Figure 1:**
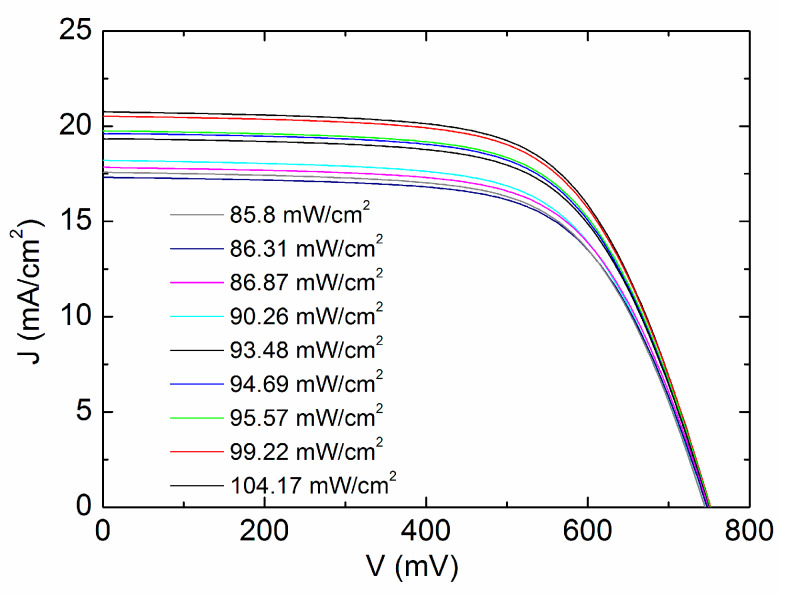
J–V curves between 85.8 mW/cm^2^ and 104.17 mW/cm^2^ with cell #1.

In this study, the above algorithm was adopted, modified and generalized in the following 4 aspects for a CdTe solar cell: ① The irradiance was originally constricted to be around 100 mW/cm^2^, while we expand the intensity to the range of 0–100 mW/cm^2^. ② *J*_0_, *n*, *R_s_* and *R_sh_* were originally assumed to be constant, while we focus on parameter variations of a CdTe solar cell. As *J*_0_, *n*, *R_s_* and *R_sh_* vary little with small intensity range, we extract the cell parameters by dividing 0–100 mW/cm^2^ into small sections. Each section corresponds to constant values of *J*_0_, *n*, *R_s_* and *R_sh_* and different sections to different values. ③ The algorithm was originally based on 5 points for linear fitting, while we consider more J–V characteristics measured for different levels of illumination, such as 9 points for linear fitting in Figure 1, which apparently leads to more accurate extraction results.④ No mechanism behind the extracted parameter was investigated in the previous report, while we link them to the recombination, traps and the body resistance of a CdTe solar cell.

## 3. Discussion

The approach can be applied to determine the parameters during each intensity range. It is confirmed that the values of *J*_0_ and *n* should be first, obtained from (2) by plotting *V_oc_* versus ln(*J_SC_*–*V_OC_*/*R_sh_*) from different illumination intensities. In this study, the intensity range of 85.8–104.17 mW/cm^2^ for cell #1 is illustrated for instance, where *J*_0_ and *n* are considered to be constant. A total of 9 J–V curves are selected for the fitting as shown in Figure 1, the values of *J_SC_*, *V_OC_*, *m_sc_*^−1^ and *m_oc_*^−1^ are obtained from J–V curves and the scatter diagram of (*J_SC–_V_OC_*/*R_sh_*)^−1^ − *m_oc_*^−1^ are plotted and linearly fitted, resulting in *nkT*/*q* through the slope and *R_s_* by the intersection with the y axis. *R_sh_* is taken to be the average value of all fitted points, and thus measured to be 199,356 Ω·cm^2^.

**Figure 2:**
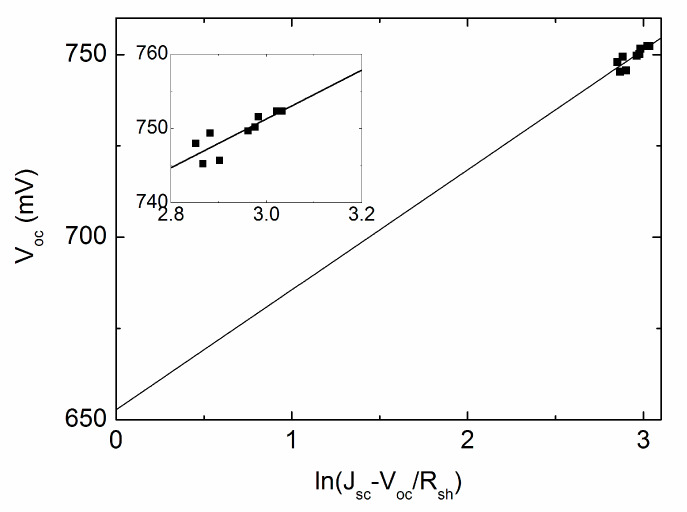
*V_oc_* versus ln(*J_SC_*–*V_OC_*/*R_sh_*) for extraction of *n* and *J*_0_ for cell #1. The Inset is an expanded view for the dotted part.

**Figure 3:**
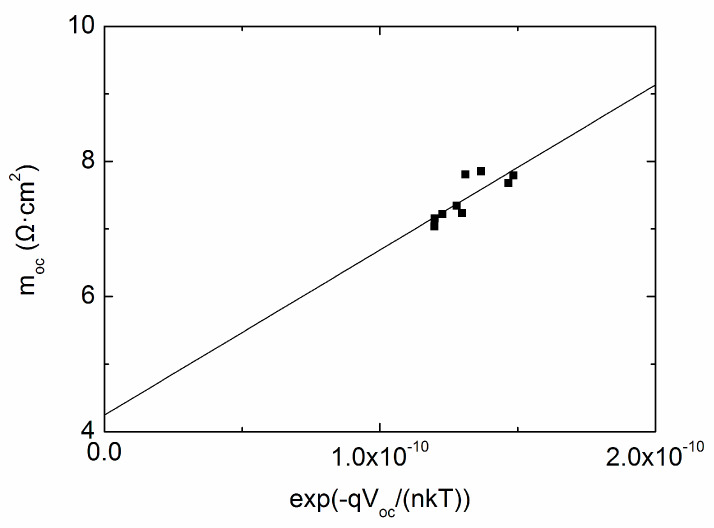
*m_oc_*^−1^ versus exp(−*qV_OC_*/(*nkT*)) for extraction of *R_s_* for cell #1.

The scatter diagram of *V_oc_* and ln(*J_SC_*–*V_OC_*/*R_sh_*) is illustrated in Figure 2 and the original extracted data from J–V curves is described in Table 1. It is shown that a straight line is fitted to the experimental data, with the slope and the intercept with the vertical axis confirmed, yielding *n* = 0.999 and *J*_0_ = 2.352 × 10^−^^9^ mA/cm^2^. The inset graph demonstrates the detailed data profile and the fitting line, it should be pointed out that the data appears to be diverse, nevertheless, the fitting is actually quite accurate with the corresponding maximum error less than 0.3%. Substituting the known *J*_0_ and *n* into Equation (3), the plot of *m_oc_*^−1^ versus exp(−q*V_oc_*/(*n*kT)) is illustrated as dots, and the subsequent linear fit is easily obtained as shown in Figure 3, *R_s_* is determined from the line intercept with the vertical axis, which is 4.244 Ω·cm^2^. The fitted values of *R_sh_*, *R_s_*, *n* and *J*_0_ are reasonably assessed to be related with the average point, meaning 92.93 mW/cm^2^ for the above discussion. It should be pointed out that the fitted values correspond to the intensity range of 85.8–104.17 mW/cm^2^, theoretically we are free to take arbitrary value between the range, if the same rule can be applied to other intensity ranges. Different data selection strategies lead to slightly different illumination values, but the same parameter variation trend in the whole illumination range. The average intensity approach is much smoother due to the reason that sometimes the testing points are not uniformly distributed. The “middle-point selection” strategy may cause parameter surge, which is explained in detail later in shunt resistance discussion.

Repeat the extraction operation under other low intensity conditions, a number of similar fitting lines are obtained at 76.16 mW/cm^2^, 63.36 mW/cm^2^, 46.38 mW/cm^2^, 32.03 mW/cm^2^ and 14.64 mW/cm^2^. It should be pointed out that the variations of the diode parameters for the irradiance lower than 20 mW/cm^2^, is partially due to the irradiance and current measurement uncertainty, as the uncertainty is relatively large under that case. In order to accurately extract *n*, *J*_0_ and *R_s_* at 14.64 mW/cm^2^, we take the largest intensity range to reduce the effect of measurement uncertainty. Finally, all parameters are extracted and listed below in Table 2.

**Figure 4:**
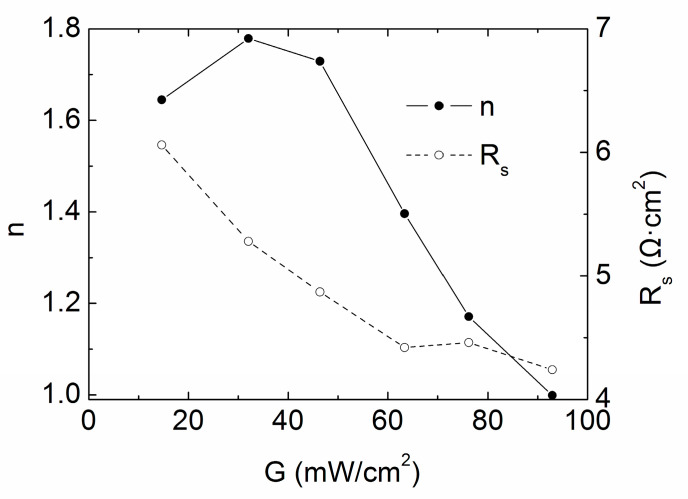
Intensity *G* versus *n* and *R_s_* under low intensity illumination condition for cell #1.

Figure 4 shows the variations of the ideality factor *n* and the series resistance *R_s_* with low solar irradiance, the former is apparently governed by recombination including bulk recombination, surface recombination and space-charge recombination, the latter is caused by bulk property and geometry. The ideality factor *n* under the standard illumination is approaching 1, indicating a nearly ideal diode, the rise in *n* with the decrease of intensity is physically suggesting a partial shift in the forward current mechanism from asymmetric recombination near the metallurgical junction, to more symmetric recombination distribution over the depletion region [21]. Therefore, the space charge recombination is more contributive at low intensities; the variation of *n* is attributed to the bulk and surface recombination mechanisms at high intensities. For *R_s_* of cell #1, it ranges between 4.24–6.06 Ω·cm^2^, denoting weak parasitic effect. As the light intensity goes down, the slight decrease of *R_s_* between 60–80 mW/cm^2^ is maybe for test deviations—or probably due to the slight decline of surface temperature, although fixed to be constant. The sharp rise of *R_s_* below 60 mW/cm^2^ is because of reduced carriers in the absorption layer. Specifically, the photo-generated carriers decline and the body resistance of CdTe absorption layer rise, while CdTe layer makes a major contribution to the variation of series resistance of the whole cell, therefore *R_s_* increases.

**Figure 5:**
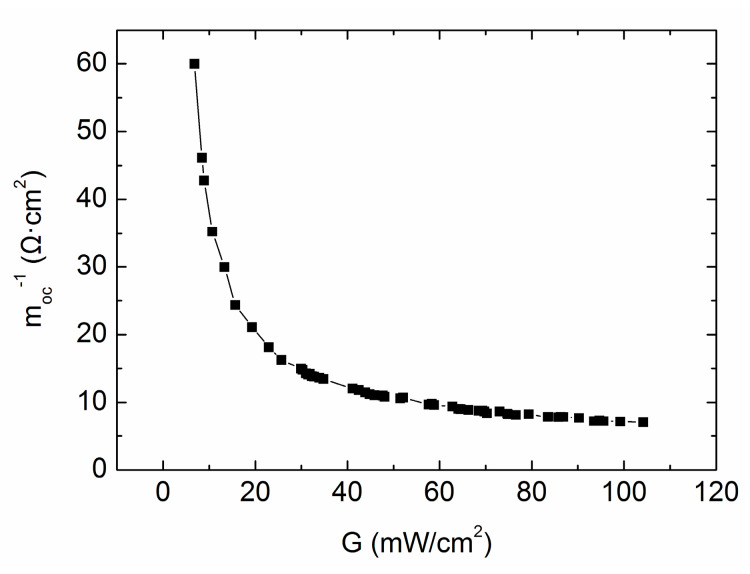
Characteristics of *m_oc_*^−1^ versus *G* for all 54 testing points with cell #1.

Next *m_oc_*^−1^ as a function of the irradiation intensity *G* is considered, as shown in Figure 5 with 54 points as the tested values. It is seen that with the decrease of *G*, *m_oc_*^−1^ rises exponentially. If we approximately regard *m_oc_*^−1^ to be *R_s_*, the curve in Figure 5 is consistent with our traditional intuition of *R_s_* versus *G* and is exactly the same as the report of Shen [7]. But the true deviation of *R_s_* is much smoother, according to our result in Figure 4, and sometimes the slight drop of true *R_s_* can be found even with the decreased illumination intensity.

**Figure 6:**
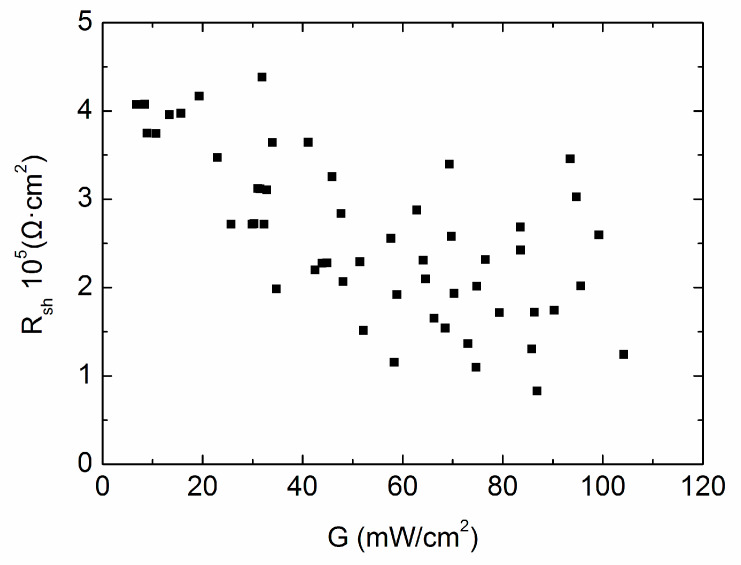
Shunt resistance *R_sh_* versus the illumination intensity *G*, including all 54 testing points.

**Figure 7:**
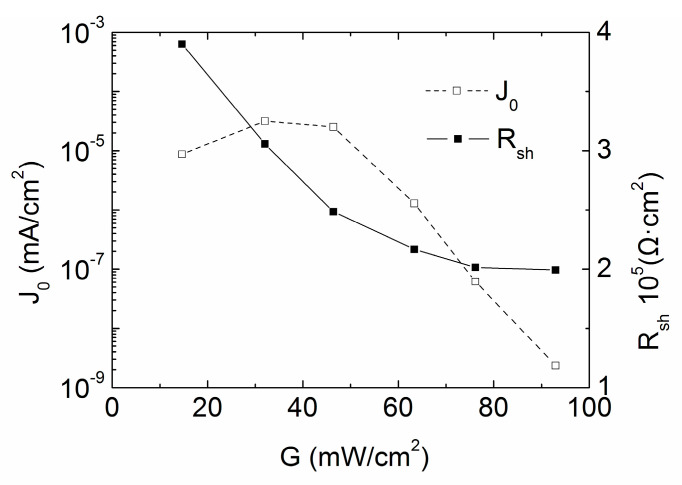
Intensity *G* versus *J*_0_ and *R_sh_* under low intensity illumination condition for cell #1.

Figure 6 demonstrates 54 shunt resistance values from all experimental J–V data, by differentiating J–V curves at short circuit and then making a reciprocal transformation. Apparently, the shunt resistance exhibits poor stability. If we take the “middle point selection” strategy representing every 9 fitting points to determine *R_sh_*, for example, *R_sh_* at 93.48 mW/cm^2^ denotes the shunt resistance at 104.17 mW/cm^2^, 99.22 mW/cm^2^, 95.57 mW/cm^2^, 94.69 mW/cm^2^, 93.48 mW/cm^2^, 90.26 mW/cm^2^, 86.87 mW/cm^2^, 86.31 mW/cm^2^ and 85.8 mW/cm^2^, it probably causes *R_sh_* to oscillate up and down within the whole intensity range, while it is far from the truth. If we take an average value of every 9 points to smooth the *R_sh_* deviation, a clear monotonous increase of *R_sh_* can be seen with the decrease of *G*, as shown in Figure 7.

Figure 7 shows the variations of the shunt resistance *R_sh_* and the reverse saturation current density *J*_0_ with low solar irradiance. *R_sh_* exceeds 100,000 Ω·cm^−2^, the defect caused current model should be employed to explain the variations, *R_sh_* is so high that the cell is well designed. With a decreased light intensity, the photo-generated carriers and hence the captured carriers also decreases, indicating the increase of *R_sh_* [22].

The reverse saturation current density relies on space charge recombination, bulk recombination, and surface recombination. The principles are as follows: (1) If the light intensity is low, the space charge recombination is significant, and the reverse saturation current density is large; (2) If the light intensity approaches 1 sun, the main contribution is from the bulk recombination and the surface recombination, and the corresponding current density is thus relatively low; (3) Less generation of carriers causes *J*_0_ to drop. Therefore, *J*_0_ monotonously increases with the irradiation intensity decreasing from about 1 sun to 30 mW/cm^2^, as recombination mechanisms dominate *J*_0_. With the light intensity lower than 30 mW/cm^2^, principles (1) and (3) take effect. When the two contributions are balanced, *J*_0_ keeps nearly constant, when principle (3) overlaps (1), the cell starts to cut *J*_0_. It is also the reason that *n* in Figure 5 reduces its value below 30 mW/cm^2^.

Next the cell with relatively low *R_sh_* is investigated. The *J*–*V* curve measurements under low irradiance intensities are carried out by a 3-A solar simulator (Crowntech) and Keithley 2400. The following figures are the data extracted by the same approach.

**Figure 8:**
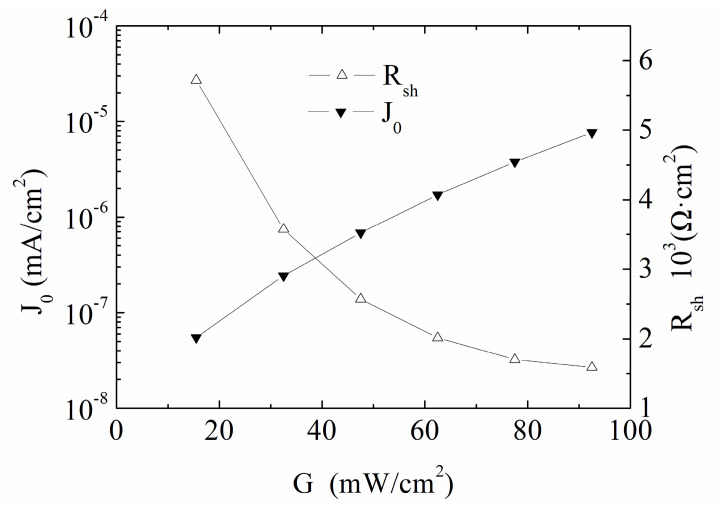
Intensity *G* versus *J*_0_ and *R_sh_* for the CdTe solar cell #2 under low intensity illumination condition.

From Figure 8, it is seen that the shunt resistance is 10 times lower than that of cell #1, the traps plays a more significant role than that of cell #1. For cell #2, with the decrease of the irradiance intensity, the captured carriers decreases due to the decreased photo-generated carriers, leading to the increase of the shunt resistance, showing an exponential tendency. The reverse saturation current density is continuously cut down due to the non-stop shifting from symmetric recombination to asymmetric recombination, which is quite different from that of cell #1.

**Figure 9:**
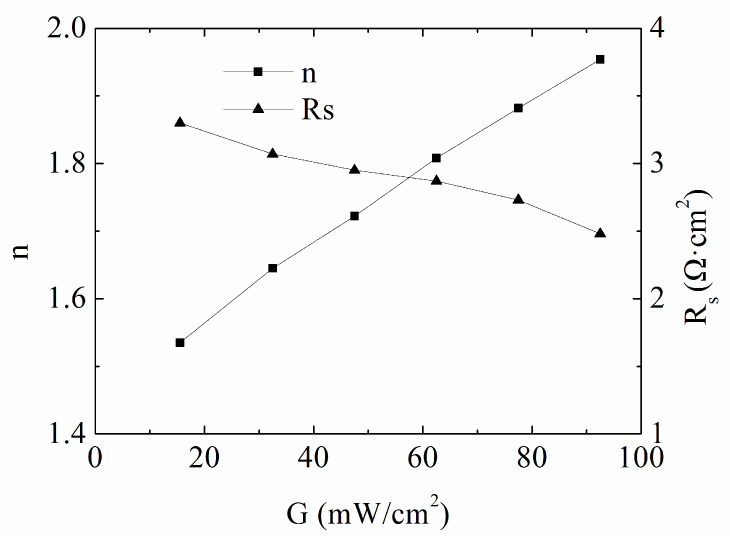
Intensity *G* versus *n* and *R_s_* for the CdTe solar cell #2 under low intensity illumination condition.

From Figure 9, the ideality factor is nearly 2 under standard irradiation condition, and drops to 1.535 with the light intensity of 17.3 mW/cm^2^, indicating the same recombination mechanism as that of the reverse saturation current density. The parasitic effect is weak with the series resistance of 2.48–3.3 Ω·cm^2^, also showing a well-fabricated cell. The carrier number lowers with decreased light intensity, resulting in a slight rise of the bulk resistance, and hence, the series resistance is on the increase.

**Figure 10:**
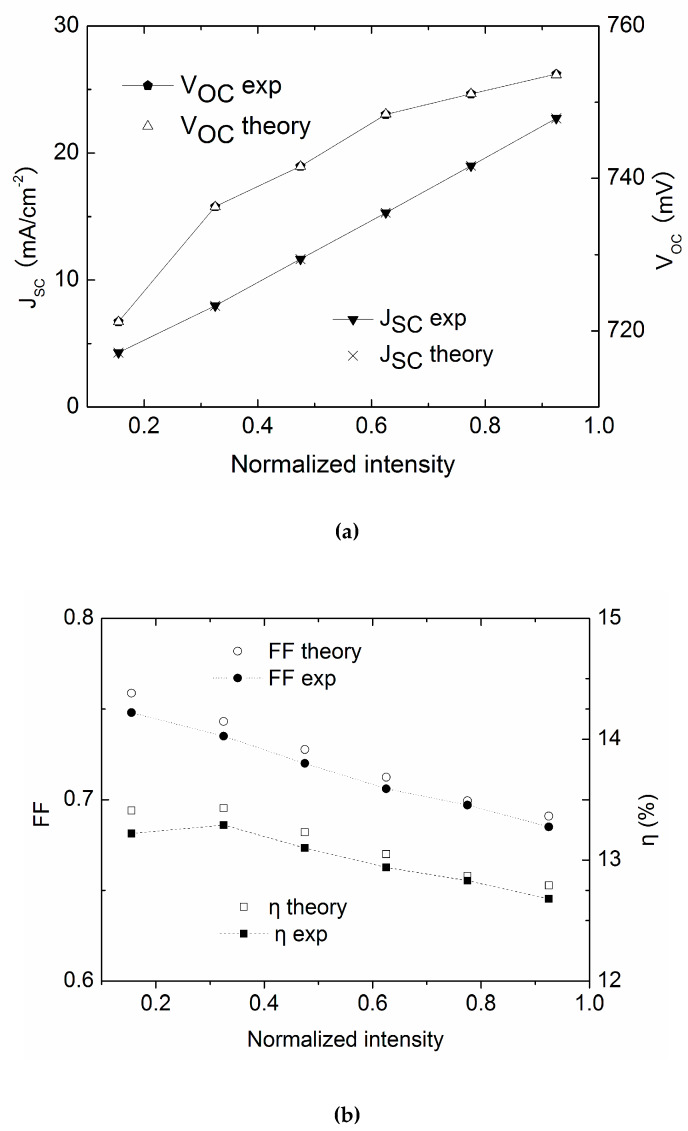
Comparison of theory and experimental values for the CdTe solar cell #2, (**a**). Jsc and Voc; (**b**) FF and η.

In order to verify the algorithm, the extracted parameters are substituted into the one-diode model, thus obtaining theoretical values of *V_oc_*, *J_sc_*, *FF* and *η*. The values are then compared with the experimental data, as shown in Figure 10a,b. Apparently, the deviations are minor, verifying the validity of the modified method.

## 4. Conclusions

In conclusion, the diode parameter variations of CdTe solar cells were investigated under low irradiance intensity. Parameters *R_sh_*, *R_s_*, *J*_0_ and *n* were regarded as constant within a narrow intensity range, and extracted based on linear fit, which makes use of a number of current curves. For cell #1 with higher *R_sh_*, with the decrease of the light intensity, *R_sh_* grew exponentially. Both *n* and *J*_0_ were determined by space charge recombination, bulk recombination and surface recombination. The first one played a leading role for lower intensity, while the rest two exhibited larger contributions if the irradiance approached STC. *R_s_* showed a steady increase for the drop of carriers in the absorber layer with a decreased intensity. For cell #2, *R_sh_* rose sharply due to the decline of trapped carriers in defect regions for *R_sh_* > 1000 Ω·cm^2^. An opposite trend of *J*_0_ for two cells was illustrated by the well-documented discussion, and so was *n*. But *R_s_* showed a similar increase in two cells for the reason of the drop in the absorber layer. The research of the CdTe solar cell layer investigation under low illumination intensity provides constructive guidelines for the future design and fabrication of CdTe solar cells.

## Figures and Tables

**Table 1:** Experimental parameter values extracted from Figure 1 and employed in Figure 2.

*G* (mW/cm^2^)	*J_sc_* (mA/cm^2^)	Voc (mV)	m_oc_^−1^ (Ω·cm^2^)	R_sh_ (Ω·cm^2^)
104.17	20.77	752.3	7.04	124,268
99.22	20.51	752.3	7.15	259,838
95.57	19.76	751.6	7.22	201,739
94.69	19.61	750.2	7.34	302,873
93.48	19.33	749.7	7.23	345,838
90.26	18.20	745.7	7.58	174,155
86.87	17.85	749.4	7.81	83,104
86.31	17.32	748	7.85	171,989
85.8	17.58	745.3	7.79	130,401

**Table 2:** Extraction of the one-diode model parameters of the CdTe solar cell #1.

*G* (mW/cm^2^)	*n*	*J*_0_ (mA/cm^2^)	*R_s_*(Ω·cm^2^)	*R_sh_*(Ω·cm^2^)
92.93	0.999	2.35× 10^−9^	4.24	73,251
76.16	1.171	6.18× 10^−8^	4.46	72,367
63.36	1.396	1.29× 10^−6^	4.42	82,882
46.38	1.729	2.52× 10^−5^	4.87	74,171
32.03	1.779	3.16× 10^−5^	5.28	67,784
14.64	1.645	8.74× 10^−6^	6.06	68,795
